# Phytochemical Investigations on Chemical Constituents of Achillea tenuifolia Lam

**Published:** 2014

**Authors:** Shirin Moradkhani, Farzad Kobarfard, Seyed Abdol Majid Ayatollahi

**Affiliations:** a *Department of Pharmacognosy and Biotechnology, School of Pharmacy, Hamadan University of Medical Sciences, Hamadan, Iran. *; b *Department of Medicinal Chemistry, School of Pharmacy, Shahid Beheshti University of Medical Sciences, Tehran, Iran. *; c *Phytochemistry Research Center, Shahid Beheshti University of Medical Sciences, Tehran, Iran. *; d *Department of Pharmacognosy and Biotechnolgy, School of Pharmacy, Shahid Beheshti University of Medical Sciences, Tehran, Iran. *

**Keywords:** *Achillea tenuifolia*, Asteraceae, Eupatorine, Lupeol, Daucosterol

## Abstract

*Achillea tenuifolia *Lam*. *(Asteraceae) afforded a methanolic extract from which after fractionation in solvents with different polarities, two known flavones 3’, 5- dihydroxy- 4’, 6, 7- trimethoxy flavone (eupatorine, compound 3), 5- hydroxy- 3’,4’, 6, 7- tetramethoxyflavone (compound 4), besides stearic acid (compound 1), lupeol (compound 2), daucosterol (β- sitosterol 3-O- β- D- glucopyranoside, compound 5), 2, 4- dihydroxy methyl benzoate (compound 6) were isolated for the first time. The structure of isolated compounds was elucidated by means of different spectroscopic methods such as UV, IR, Mass and 1H- NMR (1D and 2D) and 13C-NMR. For further confirming the structures of isolated compounds, comparison of the spectral data of them with those reported in the litratures have been done.

## Introduction

The genus *Achillea*(Asteraceae) comprises 115 species in the world which nineteen are present in Iran (1). The aerial parts of different species of the genus *Achillea *are widely used in folk medicine due to numerous pharmacological properties, such as anti-inflammation (2), antispasmodic, cytotoxic, antioxidant, antibacterial (3), antiplatelet aggregation (4). *Achillea tenuifolia *Lam. is a perennial herb distributed in western and northern regions of Iran (5). From a phytochemical point of view the following compound classes were identified in *Achillea *species: terpenoids, flavonoides, fatty acids, alkanes, lignanes and a few other types of compounds. Phytochemical studies of this plant have been initiated in view of the genus medicinal importance and the fact that the chemistry of *A.tenuifolia *concerned only the composition of the essential oil (6, 7) and fatty acid profile(8) of the species. In our previous work on *A.tenuifolia *(9), isolation of 5- hydroxy, 4’, 6, 7- trimethoxyflavone (salvigenin), β – sitosterol, methyl- gallate have been reported and metal- chelation activity of salvigenin has been investigated. 

## Experimental

*General experimental procedures*

The FT-IR spectra were recorded on a vector 22 instrument. The ^1^H-NMR was recorded on a Bruker AM 300, 400 and AM X 500 NMR (Avance) instruments using the UNIX data system at 300, 400 and 500 MHz, respectively. The ^13^C-NMR spectrum was recorded at 75, 100 and 125 MHz, respectively using CDCL_3_, CD_3_OD and C_5_D_5_N as solvent. ^1^H-^13^C HMBC and HMQC were recorded as mentioned above. EI-MS spectra were recorded on a Finnigan MAT 312. HR-EIMS were carried out on Jeol JMS 600 mass spectrometer. Column chromatography was carried out on silica gel (M&N), 70-230 and 230-400 meshes. All solvents and chemical reagents were purchased from Merk (Darmshtot, Germany). Compounds on the TLC were detected at 254 and 366 nm and by ceric sulphate as spraying reagent.

*Plant material*

The aerial flowering part of *Achillea tenuifolia *(Asteraceae) was collected in May 2008, from populations growing in Zanjan province, Iran. The plant was identified in Department of Pharmacognosy, School of Pharmacy, Shahid Beheshti University of Medical Sciences, Tehran, Iran. A voucher specimen (NO.487) was deposited in the herbarium of the above mentioned college.

*Extraction and isolation*

The dried aerial parts of *A.tenuifolia*(4 Kg) were extracted by maceration with methanol (3×15 L) at room temperature, three times, each time three days. The methanolic extract was evaporated under reduced pressure to give a dark residue (300 g), which was suspended in water and defatted with petroleum ether. The defatted aqueous extract successively fractionated with dichloromethane, *n*-butanole (3 times each). The dichloromethane fraction (50 g) was subjected on a silica gel column chromatography using hexane with increasing gradient of EtOAC up to 100% and followed by methanol to give ten fractions.

Fraction 1 (hexane eluate) was subjected on silica gel column (Hex: CHCl_3_) to yield 4 sub fractions. Sub fraction B purified by preparative TLC with the system of hexane: EtOAC (8.5:1.5) to give compound 1.

Fraction 2 (hexane:EtOAC = 9.5 : 0.5 eluate )was subjected to silica gel column chromatography, using hexane: CHCl_3_ to give three sub fractions (M- O). Sub fraction N (hexane: CHCl_3_ = 6: 4 eluate) was further purified by recrystallization from MeOH to yield compound 2 .

Fraction 6 (hex: EtOAC=8:2) was rechromatographed on silica gel column (hexane: aceton=6:4) to render 8 sub fractions (a-h).Subfractions 6c, 6f was further separated on preparative TLC to yield compounds 3, 4 using hexane: Me_2_CO, hexane: EtOAC as mobile phase, respectively.

Fraction 9 (EtOAC: MeOH= 9.5:0.5 eluate) was loaded on silica gel column using hexane: CHCl_3_: MeOH (2.5:7.5:0.5) as mobile phase and afforded compound 5.

**Figure 1:**
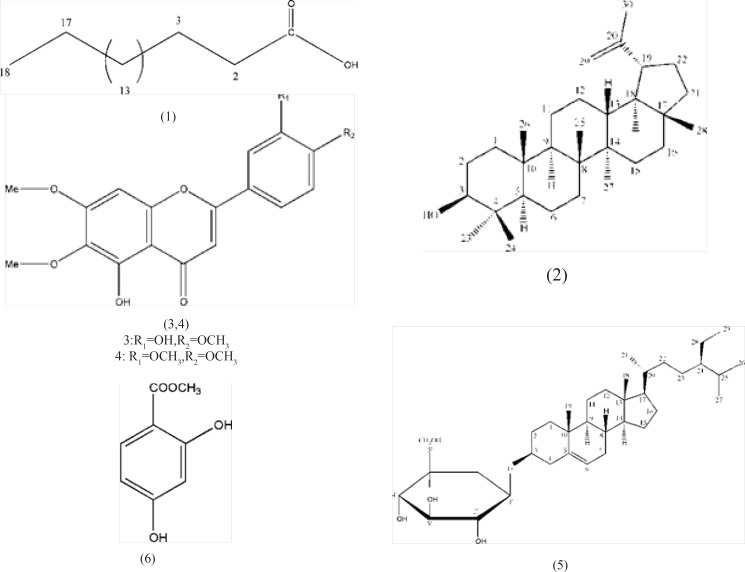
Structures of isolated compounds

Finally fraction eluted with EtOAc: MeOH (9:1) was subjected on silica gel column using Me_2_CO: MeOH as mobile phase. From fraction eluted with Me_2_CO: MeOH (9.5: 0.5) obtained compound 6.The structure of all compounds have been shown in Figure 1.

Stearic acid (compound 1): White powder(10 mg); m.p. 70 °C;HR EI/MS: 284.2722 (calcd. 284.2715 For C_18_H_36_O_2_);EI/MS: m/z(rel.%): 284(4.7), 256(25.95), 213(11.49), 185(14.45),171(14.84), 157(17.15), 143(11.18), 129(43.89), 115(17.54), 101(11.75), 87(26.50), 73(100);^1^H- NMR(CDCl_3_,300MHZ):δ=2.17(2H, t, J=7.5HZ,H-2), 1.49(2H, m, H-3), 1.16(br s, CH_2_), 0.76(3H, t, J=7HZ, H-18).

Lupeol (compound 2): Colorless crystals (20 mg); m.p. 214°- 217 °C; IR (KBr)υ_max_cm^-1^: 3400, 2950, 2890, 1510, 1360;HR EI/MS m/z: 426.6998(calcd. 426.6989 for C_30_H_50_O);EI/MS: m/z(rel.%): 426(24.77), 411(5.73), 393(2.02), 357(2.10), 302(4.30), 257(6.61), 229(6.83), 218(9.66), 207(43.76), 203(37.62), 189(57.00), 161(22.52), 121(51.33);^1^H-NMR(300MHZ, CDCl_3_): δ= 0.70(3H, s, H-24), 0.8(3H, s, H-28), 0.83(3H, s, H-25), 0.94(3H, s, H-27), 1.00(3H, s, H-23), 1.01(3H, s, H-26), 1.65(3H, s, H-30), 3.15(1H, dd, J=5.1, 11.1HZ, H-3), 4.53 (1H, br s, H-29), 4.63(1H, br s, H-29´).

3’, 5- dihydroxy- 4’, 6, 7- trimethoxyflavone (Eupatorine, compound 3): yellow solid (10 mg); m. p. 192 °C; Uv: λ_max_(MeOH): 273, 340; IRυ_max_ (KBr)cm ^-1^: 3448, 1650, 1603, 1457,1270,1120,1013,840;HR EI/MS m/z: 344.0896(calcd.344.0890for C_18_ H_16_ O_7_,);FAB MS [M+1]^+^:345,[M-1]^+^:343;EI-MSm/z(rel.int.): 343.8(35.13), 328(30.38), 313(100.00), 300(11.27), 196 (9.44), 180.8(12.45), 152.8(52.66), 148(14.45), 133(13.85);^1^H&^13 ^C-NMR: see Table 1.

**Table1:** ^1^H&^13^C- NMR assignments for the flavones 3-4.

**Pair proton ** **or carbon**	**5- hydroxy- 3´, 4´, 6, 7-tetramethoxy flavone, ** ^1^ **H- NMR,400MHZ,CDCl**_3_	**Eupatorine, ** ^1^ **H-NMR,400MHZ,CDCl**_3_	**Eupatorine, ** ^13^ **C-NMR, 100MHZ,CDCl**_3_
2			163.6
3	6.87	6.55	104.4
4			182.6
5			156.2
6			132.5
7			158.2
8	6.67	6.52	94.8
9			107.0
10			152.1
1´			123.7
2´	7.51(d,2.0HZ)	7.44(d,2.1)	113.2
3´			146.4
4´			151.2
5´	7.12(d,8.4HZ)	6.91(d,8.8HZ)	111.7
6´	7.57(dd,8.4,2.0HZ)	7.38(dd,8.8,2.1HZ)	118.8
6-OMe	3.79	3.89	55.9
7-OMe	3.98	3.94	60.9
4´-OMe	3.86	3.97	56.2
3´- OMe	3.95		

5- hydroxy- 3’, 4’, 6, 7- tetramethoxyflavone (compound 4):pale yellow crystals (10.5 mg); m. p. 195 °C; Uv:λ_max_(MeOH):270,340;IRυ_max_ (KBr)cm^-1^:3530,1660,1605,1460,1273,1130,840,800; HR EI/MS m/z: 358.1047 (calcd.358.1053 for C_19_H_18 _O_7_,);FAB MS [M+1]^+^:359,[M-1]^+^:357; EIMSm/z(rel.int.): 358(57.04), 343(50.2), 328(100.00), 313(98.6), 299(19.75), 285(32.8), 196(20), 181(30.5), 162(25.25), 153(80);^1^H- NMR: see Table 1.

Daucosterol(β- sitosterol 3-O- β- D- glucopyranoside, compound 5): White powder (25 mg); m. p. 278°- 282 °C;[α]: -14.5°; IR(KBr)υ_max_: 3460, 3050, 1650 cm^-1^;HR EI/MS m/z : 576.4386 (calcd.576.4389for C_35_H_60_O_6_); FAB MS[M-1]^+^: 575; EI/MS m/z(rel.%): 414(8.4), 399(8.1), 396(100.0), 381 (14.6), 329(4.5), 303(6.5), 275(9.9), 273(4.9), 255(20.2);^1^H-NMR(C_5_D_5_N, 500MHZ): δ= 0.64(3H, s, H-18), 0.83(3H, d, J=7.0HZ, H-27), 0.86(3H, d, J=7.0HZ, H-26), 0.92(3H, s, H-19), 0.96(3H, d, J=6.5HZ, H-21), 3.97(1H, m, H-3), 4.27-4.58(m, Glc-H), 5.04 (1H, d, J=8.0HZ,H-1´).

2,4-dihydroxy-methyl benzoate (compound 6):Brown solid (12 mg); m. p. 88°- 95 °C; HR EI/MS m/z 168.0417 (calcd.168.0423 for C_8_H_8_O_4_); EI/MS m/z(rel.%): 168.0 (100.00), 152.9 (73.29), 136.0 (25.90), 125.0(4.76), 107.9 (16.28), 97.0 (48.67), 84.9 (48.37);^1^H-NMR (CD_3_OD, 500MHZ): δ= 3.87(3H, s, OMe), 6.77 (1H,d,J=7.5HZ,H-5), 7.50 (1H, d, J=7.5HZ, H-6), 7.57(1H, s, H-3).

## Results and Discussion

Chromatographic separation of the dichloromethane fraction of *A. tenuifolia *methanolic extract led to the isolation of two flavones, as well as phytosterols, triterpenoid, fatty acid and derivative of resorcylic acid .compouds 1, 2, 5, 6 were identified as stearic acid, lupeol, daucosterol, 2,4-dihydroxy-methyl benzoate by comparison of their spectral data with those reported in the litratures (10, 11). The structures of isolated flavones were identified by interpretation of their MS,NMR,IR,UV spectra as well as by comparison of their spectral data with those reported in the litratures (12-14).

Compound 3 was found to be 3´, 5-dihydroxy-4´ ,6 ,7-trimethoxyflavone. The HREI- MS of compound 3 showed the [M]^+^ at m/z 344.0896 in agreement with the molecular formulae C_18_ H_16 _O_7_ ,corresponding to eleven degrees of unsaturation. The FAB MS [M+1]^+ ^spectra confirmed the molecular weight at 345. The EI-MS showed fragments at m/z 181 & 148,belonging to retro-Diels-Alder cleavage,which showed three oxygenated substituents in the Ring A, one hydroxyl and one methoxyl group in the Ring B.UV absorption maxima at 273 and 340 nm confirmed the presence of flavone moiety. The bathochromic shifts of Band І (in MeOH) to Band Іa (in AlCl_3_/HCl) was 18 nm, which indicated a hydroxyl group at position 5 and one methoxyl group at position 6 (15). This data was further substantiated by a ^1^H-NMR signal at δ 6.55(1H,s), which is typical for the proton of flavone (H-3).The ^1^H-NMR spectrum displayed three resonances in the aromatic region at δ7.44(1H,d,J=2.4,H-2´),δ7.38(1H,dd,J=2.4,8.8,H-6´),δ6.91(1H,d,J=8.8,H-5 ´). J=2.4, 8.8 HZ showed meta and ortho couplings, respectively, which showed that there were substitution in position 3´,4´,thus H- 2´, 6´,5´ have meta and ortho couplings with each other. Also three singlets (each integrating for three protons) at δ 3.97, 3.89, 3.94 referred to three methoxyl moieties at 4´, 6, 7 which the placement of them have been confirmed by the HMBC spectrum. The singlet at δ6.52 integrating for one proton was assigned to H- 8. The ^13^ C-NMR spectra also showed the presence of 18 carbons, which were resolved through DEPT experiment as 3 methyl, 5 methine and 10 quaternary carbons. Considering all the above evidences, the structure of the compound 3 confirmed as 3´,5-dihydroxy- 4´, 6,7-trimethoxyflavone. The structural similarities between compound 3& 4 were revealed by comparison of the ^1^H-NMR spectra of these two compounds (Table 1). In the ^1^H-NMR spectrum of compound 6,the singlet belonging to H-3 (δ 6.87),H-8(δ 6.67),3´,4´-substituted B ring (δ7.51,H-2´;7.12,H-5´;7.57,H-6´) were observed. Additional singlet at δ3.95 (3H integration) was observed, which was not present in compound 3. This was further supported by differences between HREI- MS spectra of compound 3,[M]^+^ at m/z 344.0896 and compound 4,[M]^+ ^at m/z 358.1053. In the EI- MS spectrum of compound 4 besides 358 as base ion, there were two fragment ions in m/z 181,162, characteristic retro-Diels- Alder fragment ions for three oxygenated substituents in the Ring A and two methoxyl moieties in the Ring B. All these data approved the structure of compound 4 to be, 5-hydroxy- 3´, 4´, 6, 7-tetramethoxyflavone.

*A.tenuifolia *contains compounds present in the other species of the genus. The isolation of these flavones was in accordance with the results of our previous work (9) and with the literatures on the species of *Achillea *which reported that the genus *Achillea *are characterized by the predomination of flavonoids, methylated aglycones, 6-hydroxyflavones, and their O- Me ethers (16-18).
